# Molecular diagnosis of diffuse glioma using a chip-based digital PCR system to analyze *IDH*, *TERT*, and *H3* mutations in the cerebrospinal fluid

**DOI:** 10.1007/s11060-020-03682-7

**Published:** 2021-01-08

**Authors:** Yutaka Fujioka, Nobuhiro Hata, Yojiro Akagi, Daisuke Kuga, Ryusuke Hatae, Yuhei Sangatsuda, Yuhei Michiwaki, Takeo Amemiya, Kosuke Takigawa, Yusuke Funakoshi, Aki Sako, Toru Iwaki, Koji Iihara, Masahiro Mizoguchi

**Affiliations:** 1 Department of Neurosurgery, Graduate School of Medical Sciences, Kyushu University, 3-1-1 Maidashi, Higashi-ku, Fukuoka, 812-8582 Japan; 2 Department of Neuropathology, Graduate School of Medical Sciences, Kyushu University, Fukuoka, Japan

**Keywords:** Cerebrospinal fluid, Chip-based digital PCR, Diffuse glioma, ctDNA, Liquid biopsy

## Abstract

**Purpose:**

Conventional genetic analyzers require surgically obtained tumor tissues to confirm the molecular diagnosis of diffuse glioma. Recent technical breakthroughs have enabled increased utilization of cell-free tumor DNA (ctDNA) in body fluids as a reliable resource for molecular diagnosis in various cancers. Here, we tested the application of a chip-based digital PCR system for the less invasive diagnosis (i.e., liquid biopsy) of diffuse glioma using the cerebrospinal fluid (CSF).

**Methods:**

CSF samples from 34 patients with diffuse glioma were collected from the surgical field during craniotomy. Preoperative lumbar CSF collection was also performed in 11 patients. Extracted ctDNA was used to analyze diagnostic point mutations in *IDH1* R132H, *TERT* promoter (C228T and C250T), and *H3F3A* (K27M) on the QuantStudio^®^ 3D Digital PCR System. These results were compared with their corresponding tumor DNA samples.

**Results:**

We detected either of the diagnostic mutations in tumor DNA samples from 28 of 34 patients. Among them, we achieved precise molecular diagnoses using intracranial CSF in 20 (71%). Univariate analyses revealed that the World Health Organization (WHO) grade (p = 0.0034), radiographic enhancement (p = 0.0006), and Mib1 index (p = 0.01) were significant predictors of precise CSF-based molecular diagnosis. We precisely diagnosed WHO grade III or IV diffuse gliomas using lumbar CSF obtained from 6 (87%) of 7 patients with tumors harboring any mutation.

**Conclusion:**

We established a novel, non-invasive molecular diagnostic method using a chip-based digital PCR system targeting ctDNA derived from CSF with high sensitivity and specificity, especially for high-grade gliomas.

**Supplementary Information:**

The online version of this article (10.1007/s11060-020-03682-7) contains supplementary material, which is available to authorized users.

## Introduction

Molecular diagnosis of diffuse gliomas is conventionally performed using surgically obtained tumor tissues; however, the 2016 central nervous system (CNS) World Health Organization (WHO) classification defined a novel tumor [diffuse midline glioma, *H3* K27M mutation (DMG, *H3* K27M-mut)], diagnosis of which was confirmed by molecular findings [1]. A potential diagnostic issue is that biopsies need to originate from deep tissues, such as the brainstem, and surgery to obtain tissue samples for molecular diagnosis represents a high-risk intervention. Accordingly, the development of less invasive methods for detecting tumor-specific mutations is essential.

Recent progress in genetic analysis technologies such as digital PCR systems allows precise detection of genetic alterations from small samples [2, 3] and has promoted molecular-diagnostics applications that analyze bodily fluids (liquid biopsy), specifically in various cancers, as a less invasive method for diagnosis and monitoring [4, 5]. Recent reports revealed the presence of cell-free tumor (ct)DNA in the cerebrospinal fluid (CSF) obtained from patients with CNS tumors [6–8], and ctDNA is also used as a target for liquid biopsy of diffuse glioma [9–11]. This promising approach promotes the utilization of molecular findings as diagnostic markers of diffuse gliomas for monitoring disease state and ultimately the development of non-operative diagnostic procedures. In the present study, we established a novel assay that applies liquid biopsy targeting the ctDNA in CSF using chip-based digital PCR (dPCR) for detecting the glioma-specific diagnostic driver mutations such as *IDH1* R132H (*IDH1*), *H3F3A* K27M (*H3* K27M), and *TERT* promoter (p*TERT*) mutations. Additionally, we evaluated the feasibility of this assay using CSF samples from patients with diffuse glioma.

## Methods

### Study population and sample collection

Intracranial CSF samples were collected from adult and adolescent patients between July 2017 and November 2019 during surgical resection of intracranial tumors under a preoperative diagnosis of diffuse glioma. We collected 3–10 mL of CSF from the operative field immediately after opening the dura under general anesthesia to prevent contamination with DNA released from the resected tumor during surgical manipulation. We also preoperatively collected 1–6 mL of CSF by lumbar puncture from enrolled adult and adolescent patients who underwent tumor resection of the intracranial tumor under preoperative diagnosis of diffuse glioma without highly elevated intracranial pressure between March 2019 and February 2020. We excluded non-glioma patients after confirmation of pathological diagnosis.

We centrifuged CSF samples at 5000 *g* for 10 min within 1 h of collection to remove residual cells and stored them at − 80 °C as described with slight modification [12]. DNA was extracted from 1 to 3 mL CSF using the QIAamp circulating nucleic acid kit (Qiagen, Hilden, Germany) according to manufacturer instructions and eluted in a final volume of 60 µL. Quantification of cell-free DNA (cfDNA) was determined using the Qubit 2.0 fluorimeter with the Qubit dsDNA HS assay kit (Thermo Fisher Scientific, Foster City, CA, USA). Extracted cfDNA was stored at 4 °C and analyzed within 1 week.

CSF samples were obtained and analyzed in accordance with the Declaration of Helsinki, with the approval of the Ethics Committee of our institute, and with written consent from patients.

### dPCR

Genotyping of cfDNA and tumor DNA was performed using the QuantStudio^®^ 3D Digital PCR System (Life Technologies, Carlsbad, CA, USA). The DNA template, QuantStudio^®^ master mix, and the assay containing primer/probe were mixed according to the manufacturer protocol to obtain a dPCR reaction.

The TaqMan dPCR liquid biopsy assay (Life Technologies) was used for p*TERT* C228T and p*TERT* C250T analyses, and a custom TaqMan SNP genotyping assay was used for *IDH1* and *H3* K27M mutation analyses. The primer and probe sequence data for each assay are described in the Supplemental Digital Content (see Table 1 in Supplemental Digital Content). The final 14.5-µL dPCR mixture was loaded onto a QuantStudio^®^ 3D Digital PCR chip version2 and subjected to PCR amplification using the QuantStudio^®^ 3D GeneAmp PCR system 9700 (see Table 2 in Supplemental Digital Content). Thermal cycling was performed according to the manufacturer’s instructions (see Table 3 in Supplemental Digital Content). Following the reaction, data were analyzed using QuantStudio^®^ 3D Analysis Suite (v.3.1).

### Conventional genotyping of tumor-tissue DNA

We isolated and purified DNA from snap-frozen (− 80 °C) intraoperative tumor samples obtained from all patients whose CSF was analyzed, using QIAmp DNA mini kits (Qiagen). We confirmed the presence of *IDH1*, p*TERT* C228T or C250T, and *H3* K27M point mutations by high-resolution melt analysis and subsequent Sanger sequencing, as described previously [13, 14]. Loss of heterozygosity (LOH) on chromosome 1p and 19q was confirmed by a PCR-based LOH assay using microsatellite markers, as described previously [15].

### Gel electrophoresis

Tris–acetate-EDTA (pH 8.0) was used as electrophoresis buffer, and a 1.5% agarose gel was used for the electrophoresis of a sample prepared by mixing 5 µL of DNA template with 6X loading buffer (TAKARA, Shiga, Japan). Electrophoresis was performed using a Mupid R-exu system (Mupid Co., Ltd., Tokyo, Japan) at 100 V for 30 min, followed by staining with EtBr solution and detection and imaging using a Limited-STAGE-UV system (AMZ Systems Science, Osaka, Japan).

### Statistical analysis

Student’s *t* test was used to compare the DNA concentrations at different collection sites. Associations between ctDNA positivity and patient characteristics were assessed using non-parametric tests, including the Wilcoxon rank-sum test (continuous variables) or Fisher’s exact test (categorical variables). All statistical tests were two-sided, with an α value of ≤ 0.05 used to determine statistical significance. Statistical analysis was performed using JMP software (v.15.0; SAS Institute Japan, Tokyo, Japan).

## Results

### Primer design for dPCR of cfDNA

**Fig. 1:**
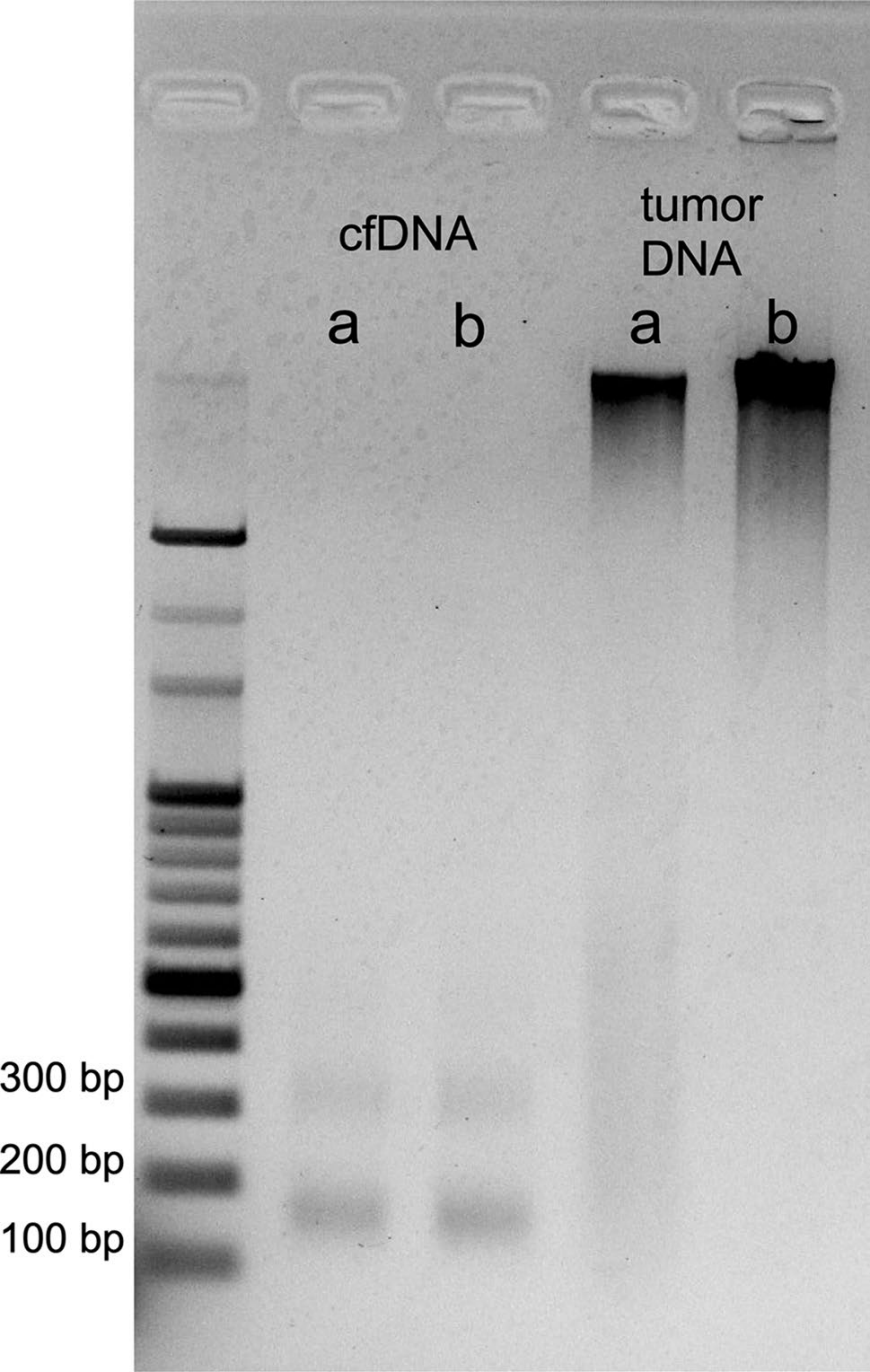
Agarose gel electrophoresis shows discrete bands at 150 and 300 bp exclusively in lanes of cell-free DNA extracted from cerebrospinal fluid of two patients (left) compared with widespread smear bands for corresponding tumor-tissue DNA (right). Two representative samples show that a high concentration of cell-free (cf)DNA can be extracted from intracranial cerebrospinal fluid. Since the concentration of cfDNA was low, we did not adjust the concentration. **a** Recurrent anaplastic oligodendroglioma in a 43-year-old female; cfDNA concentration, 4.3 ng/μL. **b** First onset of diffuse midline glioma with *H3* K27M in a 46-year-old female; DNA concentration, 4.1 ng/μL. Tumor DNA 15.0 ng/μL was loaded

To verify the reproducibility of PCR products amplified from CSF-derived cfDNA, two pairs of cfDNA and the corresponding tumor-tissue DNA obtained from different patients were subjected to gel electrophoresis (Fig. 1). Discrete cfDNA bands at 150 bp and 300 bp (the nucleosome footprint [16]) were clearly visualized exclusively in CSF cfDNA, consistent with previous reports [17]. Taken into consideration that a recent study revealed that fragment size of ctDNA is shorter than cfDNA from normal cells [18], the amplicons of significantly below 150 bp were considered appropriate to ensure stable PCR products when using CSF cfDNA as a template. Therefore, we designed the primers targeting 88- and 107-bp amplicons for *IDH1* and *H3* K27M assays, respectively (see Table 1 in Supplemental Digital Content).

### dPCR protocol for cfDNA samples

**Fig. 2:**
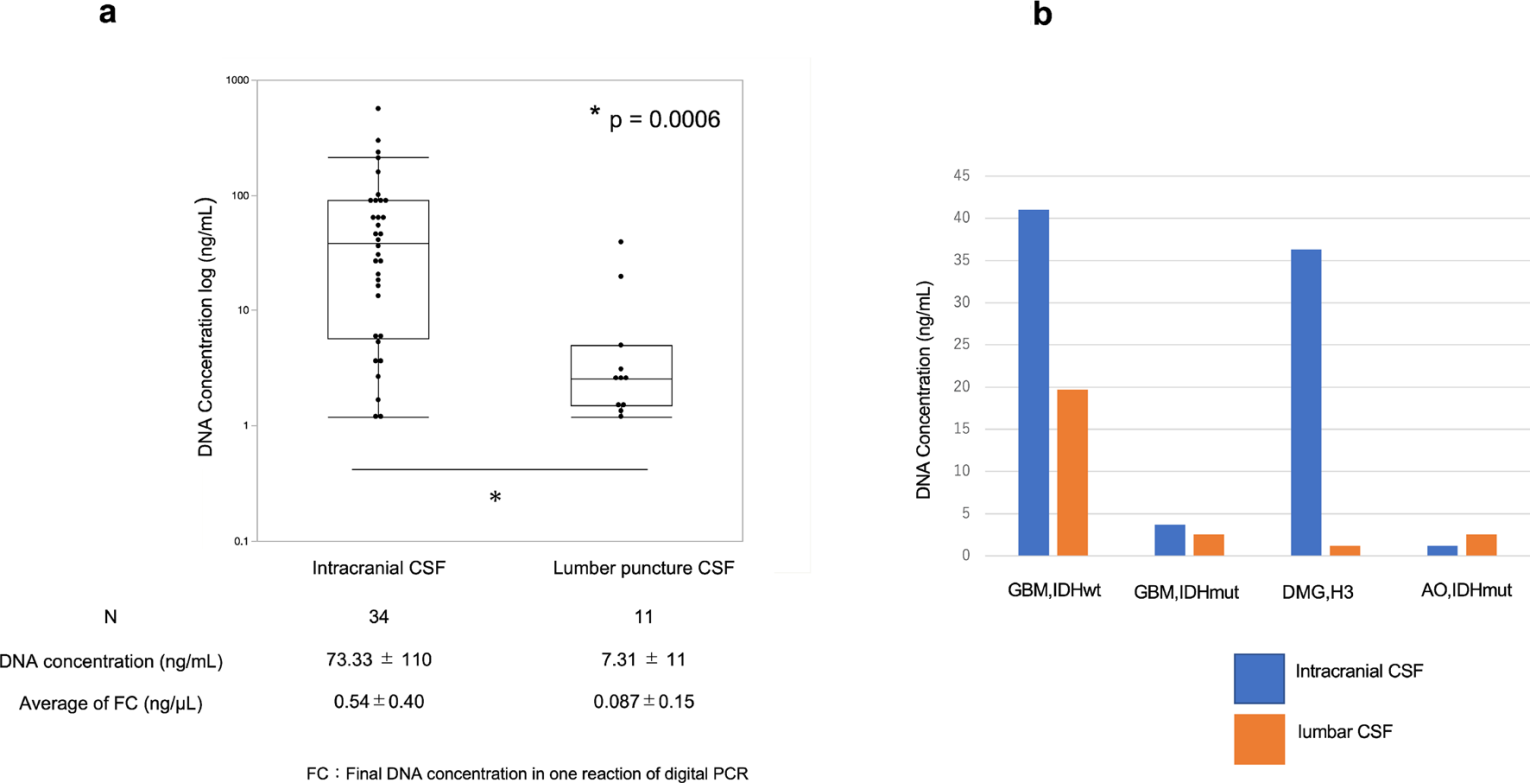
**a** Wilcoxon rank-sum test for differences in cell-free DNA concentration between the intracranial cerebrospinal fluid and that obtained after lumbar puncture. **b** Direct comparison of extracted cell-free DNA concentration between two sites in four patients

Figure 2a shows the plots of cfDNA concentrations in all collected CSF samples obtained from the intracranial operative field and lumbar puncture. The distribution of cfDNA concentrations differed significantly between the two sites, with significantly lower cfDNA concentrations obtained from CSF samples from lumbar puncture relative to those from intraoperative CSF samples (p = 0.0006). A direct comparison was performed on four patients having available paired intracranial and lumbar samples, with higher concentrations observed in intracranial samples than in lumbar CSF, except for a sample pair obtained from a patient with the highest class III cytology (Fig. 2b). Notably, the mean cfDNA concentrations in the intracranial and lumbar CSF samples were 73.33 ng/mL and 7.31 ng/mL, respectively, which were far below the recommended final concentration (1 ng/µL) for dPCR templates [3]. However, as described in the next paragraph, we managed to perform digital PCR according to our original protocol, allowing the usage of DNA samples with low concentrations (see Fig. 2a).

### Mutation status according to dPCR results

For mutation analysis, we used a pair of VIC- and FAM-labeled TaqMan probes annealing to wild type and mutant alleles, respectively [3, 19], and established the threshold values for both VIC and FAM fluorescence using control DNAs. Because the assay needs to tolerate extremely low DNA concentrations, we diluted the negative control (NC) cfDNA obtained from a patient with wild-type (WT) tumor DNA to final reaction concentrations of 1.0 ng/µL, 0.1 ng/µL, and 0.01 ng/µL each (see Fig. 1 in Supplemental Digital Content). Raw data for each reaction were retrieved and analyzed, and fluorescence values were normalized for homogeneity. We performed quadruplicate NC experiments (at 1 ng/μL) to establish threshold values to determine VIC positivity at a certain distance and calculated as the average distance from the peak value of the first distribution and the lowest value of the second distribution apart from the mode point of the VIC histogram. The threshold value for determining FAM positivity was established at a certain distance from the mode point of the FAM histogram and the maximum value of the distributions and excluding sporadic, high-fluorescence spots. The threshold values for all four mutations were established in a similar manner (see Table 4 in Supplemental Digital Content). The scatter plot of fluorescence intensity for each well showed nonspecific VIC and FAM double-positive plots, even in the NC (see Fig. 1A in Supplemental Digital Content). Theoretically, dPCR performed using very low DNA concentrations involves the distribution of template DNA at less than one copy/well, with mutant DNA detected as FAM-positive and VIC-negative plots. Therefore, we defined positive determination of the presence of mutation(s) as the existence of FAM-positive and VIC-negative plots (see Fig. 1C in Supplemental Digital Content). We performed duplicate analyses of the clinical samples and confirmed the mutation when two or more spots were detected in either of the duplicate results. An example of *IDH*-positive and -negative samples is shown in the Supplemental Digital Content (see Fig. 2 in Supplemental Digital Content).

### cfDNA analysis from intracranial CSF

Among 53 patients from whom intracranial CSF was obtained during surgery, 34 had confirmed, pathologically-diagnosed, diffuse glioma. Table 1 summarizes the characteristics of these 34 patients, who were grouped according to WHO tumor grades II, III and IV (n = 5, 13 and 16, respectively).

**Table 1:**
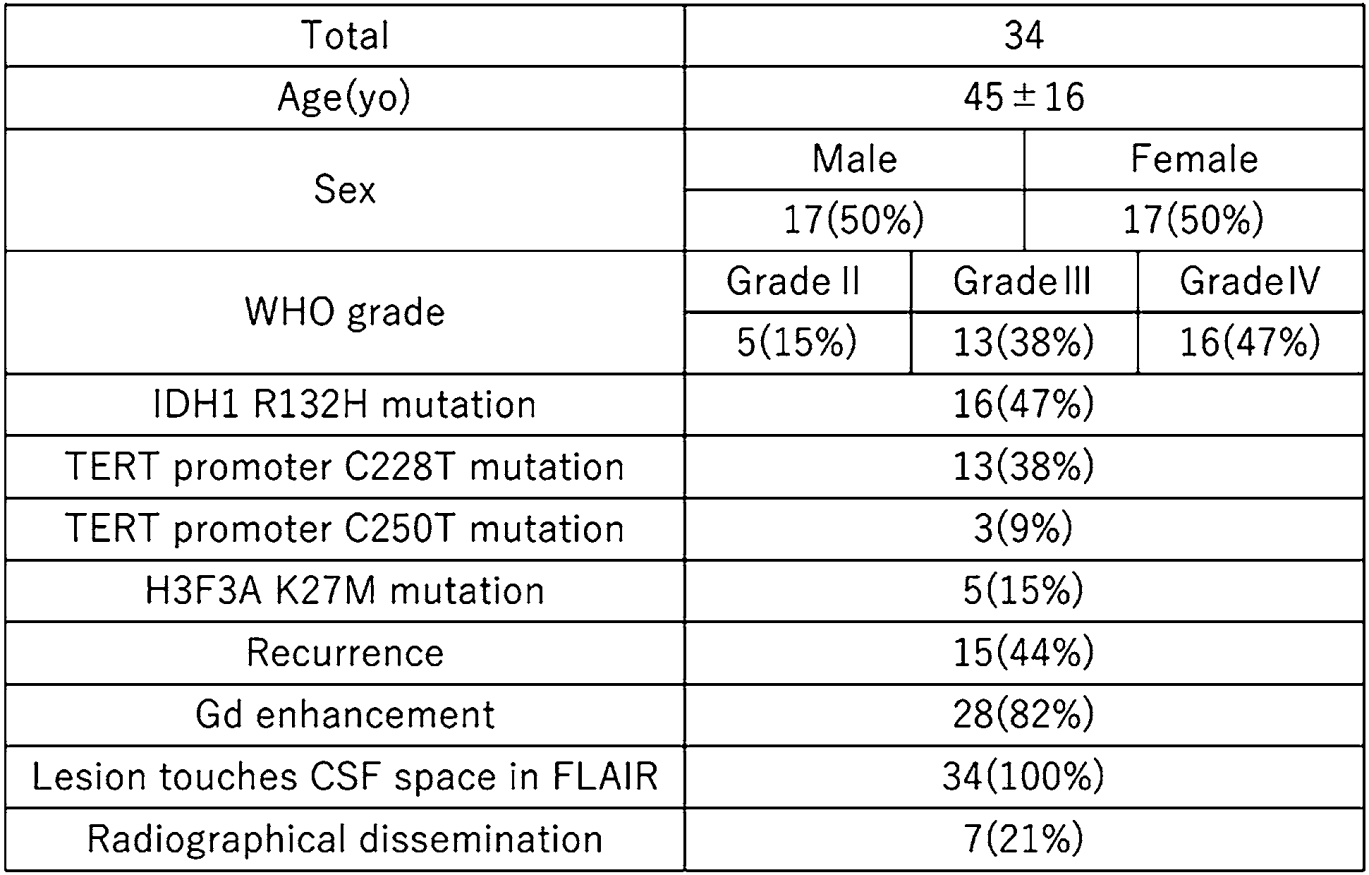
Background of patients who provided intracranial cerebrospinal fluid

Supplementary Table 5 shows a comparison of the genotype profiles between dPCR results with CSF cfDNA and Sanger sequencing with the corresponding tumor-tissue DNA. Samples without mutation(s) in the corresponding tumor were determined as WT in CSF cfDNA for *IDH1* R132H (*n* = 16), p*TERT* C228T (*n* = 22) and C250T (*n* = 32), and *H3* K27M (*n* = 32) assays. There were no false-positive findings among cfDNA results when tumor-tissue DNA results were used as controls. These results confirmed the high specificity of these assays. Because 28 (82%) of 34 tumors harbored at least one mutation in tumor-tissue DNA, we evaluated the reliability of dPCR within these results. The genotypes of all glioblastoma (GBM) samples (*n* = 7) were confirmed by using dPCR, with the *H3* K27M mutation confirmed in 4 of 5 patients with DMG, *H3* K27M-mut. *IDH* and p*TERT* co-mutations, a hallmark of oligodendroglioma [20–22], were confirmed in all eight patients with anaplastic oligodendroglioma. The remaining eight gliomas using cfDNA could not be genotyped except for an anaplastic astrocytoma with an *IDH* mutation. Univariate analyses revealed that the WHO grade (p = 0.0034), radiographic enhancement (p = 0.0006), recurrence (p = 0.0084), and Mib1 index (p = 0.01) were significant predictive factors for precise CSF-based molecular diagnosis; however, DNA concentration and radiographic dissemination were not predictive (Table 2).

**Table 2:** Univariate analysis

	Matched N = 20	Failure N =8	p
WHO grade III or IV	20	4	0.0034
Gd enhancement	20	3	0.0006
Recurrence	12	0	0.0084
Mib-1index(%)	33 ± 21	12 ± 13	0.01
Radiographical dissemination	5	1	0.64
Final concentration (ng/μ**L)**	0.58 ± 0.12	0.50 ± 0.10	0.58

### Analysis of cfDNA from CSF obtained by lumbar puncture

Of the 16 patients undergoing pre-surgery lumbar puncture, pathological diagnosis of diffuse glioma was confirmed in 11, which were enrolled for subsequent cfDNA genotyping (Table 3). Nine of 11 tumors (82%) harbored at least one mutation in tumor-tissue DNA, and genotyping results for CSF and tumor tissue matched in all WHO grade IV tumors. In the remaining five cases, accurate results were obtained for two cases: a primary anaplastic astrocytoma involving *IDH* mutation without radiographic enhancement and a recurrent anaplastic oligodendroglioma manifesting radiographic enhancement and dissemination. The results showed that 6 (87%) of 7 WHO grades III and IV gliomas were precisely diagnosed preoperatively, whereas neither of the grade II tumors could be preoperatively diagnosed. Similar to results obtained from intracranial CSF, lumbar CSF samples did not return false-positive results during genotyping. Notably, grade IV tumors were accurately diagnosed preoperatively, even in patients with cytology class I or without radiographic dissemination (see Fig. 3 in Supplemental Digital Content).

**Table 3:** Cerebrospinal fluid cfDNA derived from lumbar puncture

Case	Age	Sex	Molecular diagnosis	WHO grade	Mib-1index (%)	Primary/ recurrence	Gd enhancement	Cytology class	Radiographical dissemination	Tumor DNA				Final concen tration (ng/*μ* L)	Match
										IDH	TERT 228	TERT 250	H3 K27M		
1	43	F	GBM,IDH mut	IV	22.8	rec	+	1	+	mut	wt	wt	wt	0.06	Yes
2	74	M	GBM,IDH wt	IV	9.5	pri	+	2	−	wt	mut	wt	wt	0.13	Yes
3	53	M	GBM,IDH wt	IV	11.6	pri	+	2	+	wt	mut	wt	wt	0.54	Yes
4	84	F	GBM,IDH wt	IV	58	pri	+	2	+	wt	wt	wt	wt	0.07	−
5	33	M	GBM,IDH wt	IV	60	pri	+	2	−	wt	wt	wt	wt	0.018	−
6	18	F	DMG,H3 K27M	IV	5.4	pri	+	2	−	wt	wt	wt	mut	0.03	Yes
7	47	F	AA,IDH mut	III	50	pri	−	2	−	mut	wt	wt	wt	0.075	Yes
8	43	M	AO,IDH mut	III	22.8	rec	+	3	+	mut	mut	wt	wt	0.06	Yes
9	43	F	AO,IDH mut	III	25	pri	−	2	−	mut	mut	wt	wt	0.04	No
10	49	F	DA,IDH mut	II	1.4	pri	−	na	−	mut	wt	wt	wt	0.02	No
11	63	F	OA IDH mut	II	3.7	pri	−	2	−	mut	mut	wt	wt	0.03	No

## Discussion

In this study, we describe the establishment of a novel method for liquid biopsy targeting the ctDNA in CSF using chip-based dPCR to detect glioma-specific diagnostic driver mutations. The 2016 CNS WHO classification and subsequent cIMPACT-NOW update incorporated various glioma-specific genetic alterations in order to integrate molecular diagnoses [1, 20, 21, 23, 24]. Among these alterations, the 2016 CNS WHO classification regards the *IDH* mutation and the 1p19q co-deletion as essential markers for a molecular diagnosis of diffuse glioma, and the *H3* K27M mutation as an essential marker of pediatric glioma [1]. Additionally, p*TERT* mutation is recognized as a novel essential diagnostic marker based on its presence in GBMs and oligodendrogliomas [22]. Recent studies revealed p*TERT* mutations as the most prevalent molecular markers detected in *IDH*-WT GBMs and predictive of aggressive bioactivity of diffuse astrocytomas [25]. cIMPACT-NOW (update 3) reached a consensus on designating *IDH*-WT diffuse or anaplastic astrocytomas with p*TERT* mutation as WHO grade IV [22, 26]. In *IDH*-mutant oligodendroglial tumors, the status of 1p19q and p*TERT* are strongly correlated, with these markers interchangeable when combined with *IDH* status [20–22]. Although DMG, *H3* K27M-mut predominantly occurs in children and adolescents, high-grade thalamic gliomas from young adults frequently harbor *H3* F3A-K27M [27]. The present study of six patients with *H3* K27M-mut included three adult-onset cases. Moreover, the frequency of elderly patients with DMG, *H3* K27M-mut is notable [28, 29]. We recently described an elderly patient with the DMG, *H3* K27M-mut that mimicked a hemispheric malignant glioma [30]. Taken together, the *H3* status of an adult hemispheric diffuse glioma affecting the midline should be confirmed even in elderly patients. It is conceivable that the majority of diffuse gliomas can be molecularly diagnosed by confirming *IDH*, *H3* K27M, and p*TERT* status. In the present study, we selected these genes based on this interpretation and also their status as hotspot point mutations, which enable their detection by dPCR via design of a single TaqMan probe per mutation.

Although other liquid biopsy methods for glioma diagnosis exist, sensitivity is essential for selection of analytical methods for future clinical applications. The chip-based dPCR method presented here yielded highly sensitive results using lumbar puncture CSF, especially for diagnosis high-grade diffuse gliomas. Pentsova et al. [31] collected preoperative CSF by lumbar puncture from 53 patients with suspected brain tumors and detected ctDNA in 6 (50%) of 12 of them with diffuse glioma using next generation sequencing (NGS). Miller et al. [32] also used NGS to analyze CSF samples from patients with diffuse gliomas when a clinical event occurred after completing adjuvant therapy, and identified at least one glioma-related genetic alteration in 42 (49.2%) of 85 tumors. Although the target genes and backgrounds differed from those in the present study, our results demonstrated a higher sensitivity in the dPCR-based method than NGS-based method.

Currently, commercially available dPCR systems are theoretically divided into droplet digital (dd) PCR and chip-based dPCR. The sensitivity of ddPCR exceeds that of NGS [33]. Martinez et al. [10] tested the capability of ddPCR using cfDNA samples from 20 cases of diffuse gliomas and successfully detected ctDNAs, such as p*TERT*, *IDH*, and *H3* K27M, in 15 (88%) of 17 patients using CSF collected from lumbar puncture. This indicated that ddPCR showed comparable or higher sensitivity relative to the dPCR assay in the present study; however, Martinez et al. [10] used their method exclusively to validate an already confirmed mutation. Because we performed all four genotyping assays using a limited amount of CSF from each patient, we were forced to analyze further diluted DNAs from the originally extracted samples. Therefore, we speculate that the sensitivity of our results might have been reduced due to the study design, and that the sensitivity could have been improved by focusing on a target gene. Moreover, chip-based dPCR offers a higher degree of cost-effectiveness and simpler operation relative to ddPCR as a practical method [3, 19]. Given the aim of liquid biopsy as allowing molecular diagnosis in the absence of surgery, high specificity is required for clinical applications. These results showed no false-positive determinations of mutations using cfDNAs relative to tumor-tissue DNA, indicating the extremely high specificity of the method. Accordingly, these findings suggest the efficacy of this assay for future development of a liquid biopsy acceptable for daily clinical use.

One of the major limitations of the present study is that the reliability of our method seemed to depend on the extent of CSF involvement in tumor distribution. Therefore, our method might be most appropriate for diagnosing advanced high-grade glioma involving CSF. However, we should take into consideration that lumbar puncture is not always indicated for patients with advanced high-grade glioma, because of risk of herniation by space-occupying lesions. These issues are associated with another limitation of the present study that most of our samples were obtained by craniotomy, and lumbar CSF was collected from a limited number of our patients. We detected diagnostic mutations in 28 of 34 patients with glioma using intracranial CSF and the analyzed genes in the remaining six gliomas did not harbor any diagnostic mutations even in tumor tissues. This issue is associated with another limitation that gliomas cannot always be differentially diagnosed from non-glial tumors through genotyping of diagnostic mutations. The sensitivity of our method is lower for lumbar than for intracranial CSF is another limitation. The sensitivity of our method needs to be improved, even for lumbar CSF, before less-invasive liquid biopsies could be routinely used for diagnostic purposes.

## Conclusions

Here, we showed that molecular analysis of diffuse glioma can be performed using chip-based dPCR of ctDNA extracted from patient CSF. Lumber CSF analysis is a less invasive than conventional molecular diagnostic methods. This approach facilitates further development of novel methods of molecular diagnosis and future paradigm shifts in treatment strategies for and clinical management of glioma.

## Supplementary Information

Below is the link to the electronic supplementary material.

Supplementary material 1 (PDF 3511 kb) **Fig 1** (a) Establishment of threshold values for determining VIC- and FAM-positive results in the *IDH1* R132H assay. A negative control assay was performed with a final concentration of 1 ng/μLof the sample. The scatter plot shows bimodal VIC-negative with a positive distribution (left). We established the threshold value for determining VIC positivity at a certain distance (indicated as “X”) apart from the mode point of the VIC histogram (center). The threshold value for determining FAM positivity was set at a certain distance (indicated as “Y”) apart from the mode point of the FAM histogram (right). “X” was calculated as the average distance from the peak value of the first distribution and the lowest value of the second distribution. “Y” was determined as the distance from the mode, with the maximum value of the distributions excluding sporadic high-fluorescence spots (arrow heads) within quadruplicate negative control reactions. (b) Validation of threshold values with lower-concentration samples. We performed negative control assays with final concentrations of 0.1 ng/μL (upper) and 0.01 ng/μL (lower) samples. The results indicated no spots in the upper-left area and only sporadic spots in the upper-right area. (c) Example of a sample with a mutation. Based on our threshold values for VIC- and FAM-labeled samples, mutant spots are shown in the upper-left area (blue). **Fig 2** Representative result of chip-based digital PCR for the *IDH1* R132 mutation based on FAM- and VIC-fluorescence channels. The clustered yellow dots show empty wells. Blue dots indicate FAM-positive wells involving amplification of a mutant allele, and red dots show VIC-positive wells involving amplification of a wild-type allele. The left image shows a cluster of blue dots indicating a mutant genotype. The right image shows a result for a wild-type genotype. **Fig 3** Representative 18-year-old female patient with diffuse midline glioma, *H3* K27M-mutation (a) A hyper-intensity lesion with slight gadolinium enhancement extending from the brain stem to cerebellum (left, FLAIR; right, gadolinium-enhanced T1WI) was biopsied and diagnosed as diffuse midline glioma. (b) Hematoxylin and eosin staining revealed diffuse infiltration of glioma cells with roundish, monotonous, hyperchromatic nuclei, mainly in the subcortical white matter. (c) Immunohistochemistry revealed *H3* K27M-positive cells located sporadically among background neural cells. (d) We extracted DNA from surgically obtained tumor tissue and performed digital PCR to reveal the *H3* K27M mutation, which was not detected by Sanger sequencing (left, digital PCR; right, Sanger sequencing). (e) Digital PCR revealed the mutation in both intracranial and lumbar cerebrospinal fluids (left, intracranial result; right, lumbar result).
