# Supplementary material for: Molecular diagnosis of diffuse glioma using a chip-based digital PCR system to analyze IDH, TERT, and H3 mutations in the cerebrospinal fluid
Source: J Neurooncol. 2021 Jan 8;152(1):47–54. doi: 10.1007/s11060-020-03682-7 (PMC7910241; doi:10.1007/s11060-020-03682-7)
Supplement: Supplementary file 1 — Supplementary material 1 (PDF 3511 kb) Fig 1 (a) Establishment of threshold values for determining VIC- and FAM-positive results in the IDH1 R132H assay. A negative control assay was performed with a final concentration of 1 ng/μLof the sample. The scatter plot shows bimodal VIC-negative with a positive distribution (left). We established the threshold value for determining VIC positivity at a certain distance (indicated as “X”) apart from the mode point of the VIC histogram (center). The threshold value for determining FAM positivity was set at a certain distance (indicated as “Y”) apart from the mode point of the FAM histogram (right). “X” was calculated as the average distance from the peak value of the first distribution and the lowest value of the second distribution. “Y” was determined as the distance from the mode, with the maximum value of the distributions excluding sporadic high-fluorescence spots (arrow heads) within quadruplicate negative control reactions. (b) Validation of threshold values with lower-concentration samples. We performed negative control assays with final concentrations of 0.1 ng/μL (upper) and 0.01 ng/μL (lower) samples. The results indicated no spots in the upper-left area and only sporadic spots in the upper-right area. (c) Example of a sample with a mutation. Based on our threshold values for VIC- and FAM-labeled samples, mutant spots are shown in the upper-left area (blue). Fig 2 Representative result of chip-based digital PCR for the IDH1 R132 mutation based on FAM- and VIC-fluorescence channels. The clustered yellow dots show empty wells. Blue dots indicate FAM-positive wells involving amplification of a mutant allele, and red dots show VIC-positive wells involving amplification of a wild-type allele. The left image shows a cluster of blue dots indicating a mutant genotype. The right image shows a result for a wild-type genotype. Fig 3 Representative 18-year-old female patient with diffuse midline glioma, H3 K27M-mutation (a) A hy [file 11060_2020_3682_MOESM1_ESM.pdf]

Supplementary figures and tables

- **Molecular diagnosis of diffuse glioma using a chip-based digital PCR system to analyze *IDH*, *TERT*, and *H3* mutations in the cerebrospinal fluid**
-

- Yutaka Fujioka[1], Nobuhiro Hata[1,*], Yojiro Akagi[1], Daisuke Kuga[1], Ryusuke Hatae[1], Yuhei Sangatsuda[1], Yuhei Michiwaki[1], Takeo Amemiya[1], Kosuke Takigawa[1], Yusuke Funakoshi[1], Aki Sako[1], Toru Iwaki[2], Koji Iihara[1], Masahiro Mizoguchi[1]
-
- [1]Department of Neurosurgery, Graduate School of Medical Sciences, Kyushu University, Fukuoka, Japan
- [2]Department of Neuropathology, Graduate School of Medical Sciences, Kyushu University, Fukuoka, Japan
-
- *** Correspondence, proofs, and reprint requests to:**
- Nobuhiro Hata, MD, PhD
- Department of Neurosurgery, Graduate School of Medical Science, Kyushu University, 3-1-1 Maidashi, Higashi-ku, Fukuoka 812-8582, Japan.
- Tel: +81-92-642-5524
- Fax: +81-92-642-5526
- E-mail: hatanobu@ns.med.kyushu-u.ac.jp

Supplementary table 1

Assay data

mutation	Assay ID	product length(bp)	Forward Primer	Reverse Primer	Wild type probe	Mutation probe
IDH R132H	ANDJ4XD	88	GCAAAATCACATTATTGCCAACA	CTTGTGAGTGGATGGGTAAAACC T	AAGCATGACGACCTA T	TAAGCATGATGACCTATG
H3K27M	ANFVT27	107	CTTTGTCCCATTTTTTTCCTGTTT	GAAGCAACTGGCTACAAAAGCC	CGCACTCTTGCGAGC	CGCACTCATGCGAGC
TERT promoter mutation C228T	Hs000000092_rm	NA	NA	NA	NA	NA
TERT promoter mutation C250T	Hs000000093_rm	NA	NA	NA	NA	NA

NA: not available

Supplementary table 2

IDH1R132H mutation and H3K27M mutation

Stage 1	Stage 2		Stage 3	
96℃	56.0℃	98.0℃	60.0℃	10.0℃
10 min	2 min	30 s	2 min	∞
1x (Hold)	39x (Cycles)		1x (Hold)	

TERT promoter mutation C228T and C250T

Stage 1	Stage 2		Stage 3	
96℃	55.0℃	98.0℃	55.0℃	10.0℃
10 min	2 min	30 s	2 min	∞
1x (Hold)	54x (Cycles)		1x (Hold)	

Supplementary table 3

	1 reaction µL	Final concetration ng/µL
Digital PCR master mix v2	7.25	1x
Taqman Assay 20x (primer + probe)	0.75	1x
Nuclease free water	6.5-X	
Template DNA	X	X/14.5
Total	14.5	

X: Up to 6.5 µL

Supplementary table 4

Taqman Assay	X	Y
IDH1 R132H mutation	1200	4000
H3F3A K27M mutation	1400	4000
TERT promoter mutation C228T	350	1200
TERT promoter mutation C250T	400	2000

VIC threshold = mode + X   FAM threshold = mode + Y

Supplementary table 5
Molecular diagnosis

<table>
<tr><th rowspan="2">Molecular diagnosis</th><th rowspan="2">WHO grading</th><th colspan="4">Tumor DNA</th><th rowspan="2">N</th><th rowspan="2">matched</th><th rowspan="2">sensitivity</th></tr>
<tr><th>IDH</th><th>TERT 228</th><th>TERT 250</th><th>H3 K27M</th></tr>
<tr><td>GBM,IDH mut</td><td>Ⅳ</td><td>mut</td><td>wt</td><td>wt</td><td>wt</td><td>4</td><td>4</td><td>100%</td></tr>
<tr><td rowspan="2">GBM,IDH wt</td><td rowspan="2">Ⅳ</td><td rowspan="2">wt</td><td>mut</td><td>wt</td><td rowspan="2">wt</td><td rowspan="2">3</td><td rowspan="2">3</td><td rowspan="2">100%</td></tr>
<tr><td>wt</td><td>mut</td></tr>
<tr><td>DMG,H3K27M</td><td>Ⅳ</td><td>wt</td><td>wt</td><td>wt</td><td>mut</td><td>5</td><td>4</td><td>80%</td></tr>
<tr><td rowspan="2">AO,IDH mut</td><td rowspan="2">Ⅲ</td><td rowspan="2">mut</td><td>mut</td><td>wt</td><td rowspan="2">wt</td><td rowspan="2">8</td><td rowspan="2">8</td><td rowspan="2">100%</td></tr>
<tr><td>wt</td><td>mut</td></tr>
<tr><td>AA,IDH mut</td><td>Ⅲ</td><td>mut</td><td>mut</td><td>wt</td><td>wt</td><td>2</td><td>1</td><td>50%</td></tr>
<tr><td rowspan="2">AA,IDH wt</td><td rowspan="2">Ⅲ</td><td rowspan="2">wt</td><td>mut</td><td>wt</td><td rowspan="2">wt</td><td rowspan="2">2</td><td rowspan="2">0</td><td rowspan="2">0%</td></tr>
<tr><td>wt</td><td>mut</td></tr>
<tr><td rowspan="2">OD,IDH mut</td><td rowspan="2">Ⅱ</td><td rowspan="2">mut</td><td>mut</td><td>wt</td><td rowspan="2">wt</td><td rowspan="2">1</td><td rowspan="2">0</td><td rowspan="2">0%</td></tr>
<tr><td>wt</td><td>mut</td></tr>
<tr><td>DA,IDH mut</td><td>Ⅱ</td><td>mut</td><td>wt</td><td>wt</td><td>wt</td><td>1</td><td>0</td><td>0%</td></tr>
<tr><td rowspan="2">DA,IDH wt</td><td rowspan="2">Ⅱ</td><td rowspan="2">wt</td><td>mut</td><td>wt</td><td rowspan="2">wt</td><td rowspan="2">2</td><td rowspan="2">0</td><td rowspan="2">0%</td></tr>
<tr><td>wt</td><td>mut</td></tr>
<tr><td>GBMorAAorDA</td><td>-</td><td>wt</td><td>wt</td><td>wt</td><td>wt</td><td>6</td><td>6</td><td>100%</td></tr>
</table>

mut: mutant wt: wildtype GBM: glioblastoma DMG: diffuse midline glioma AO: anaplastic oligodendroglioma AA: anaplastic astrocytoma OD: oligodendroglioma DA: diffuse astrocytoma

Supplementary **Fig 1a**

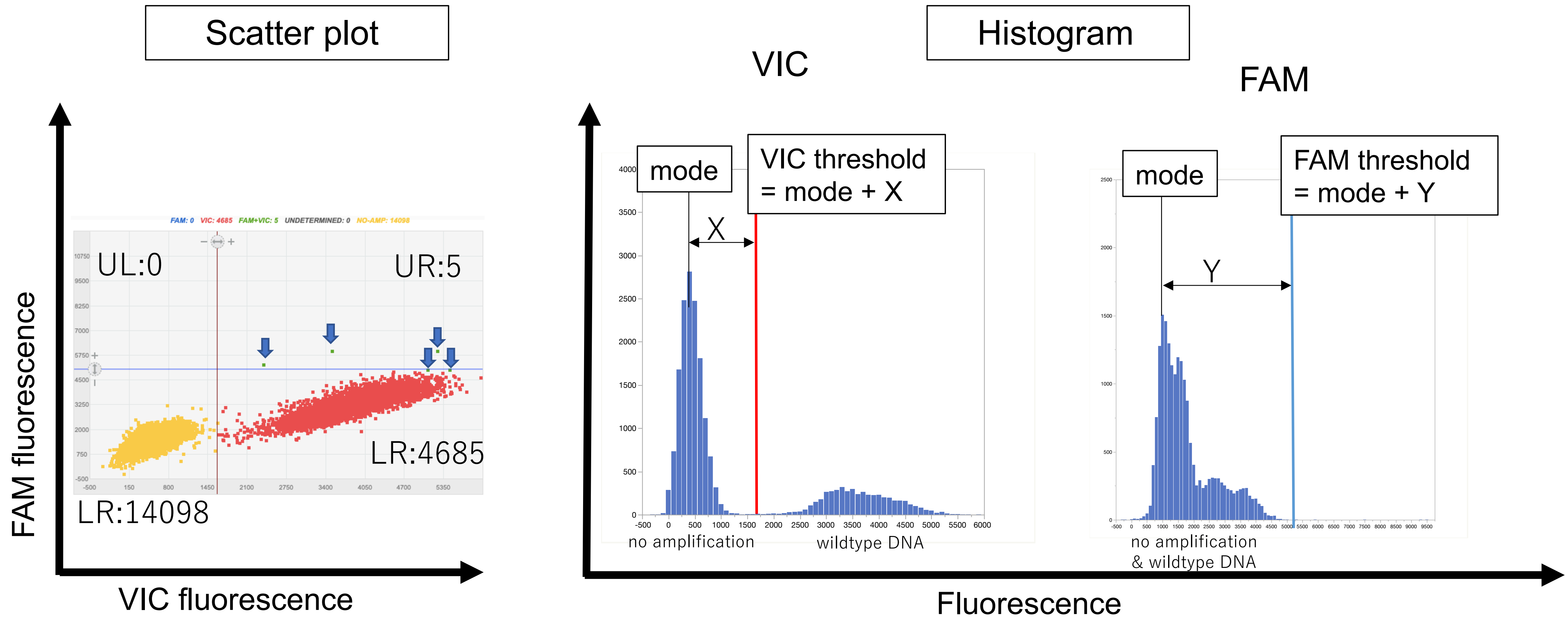

UL: Upper left
UR: Upper right
LL: Lower left
LR: Lower right

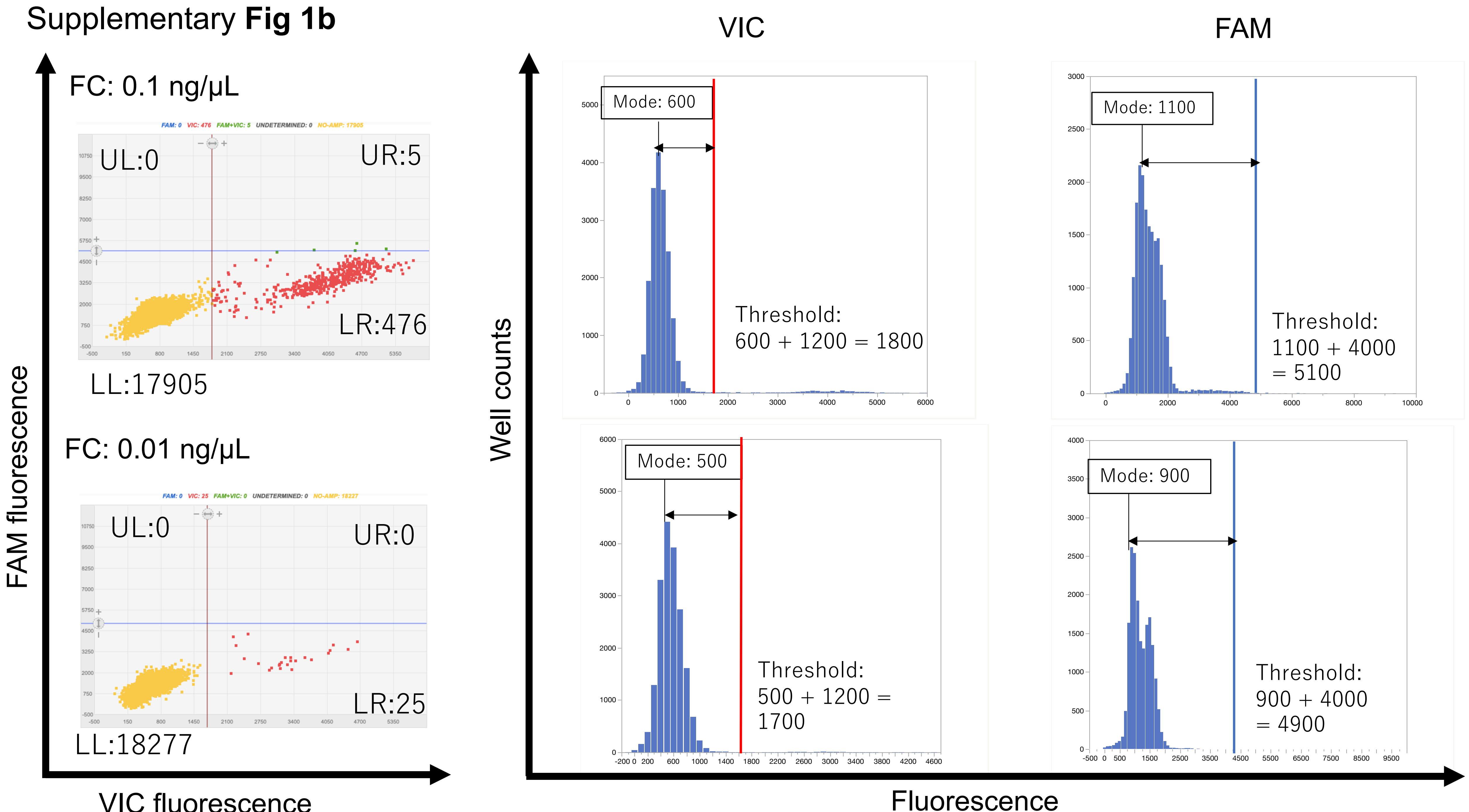

Supplementary **Fig 1b**
VIC
FAM
FC: 0.1 ng/μL
FAM: 0 VIC: 476 FAM+VIC: 5 UNDETERMINED: 0 NO-AMP: 17905
UL:0
UR:5
LR:476
LL:17905
FC: 0.01 ng/μL
FAM: 0 VIC: 25 FAM+VIC: 0 UNDETERMINED: 0 NO-AMP: 18227
UL:0
UR:0
LR:25
LL:18277
FAM fluorescence
VIC fluorescence
Well counts
Mode: 600
Threshold:
600 + 1200 = 1800
Mode: 1100
Threshold:
1100 + 4000
= 5100
Mode: 500
Threshold:
500 + 1200 =
1700
Mode: 900
Threshold:
900 + 4000
= 4900
Fluorescence

Supplementary **Fig 1c**

IDH1 R132H mutation assay

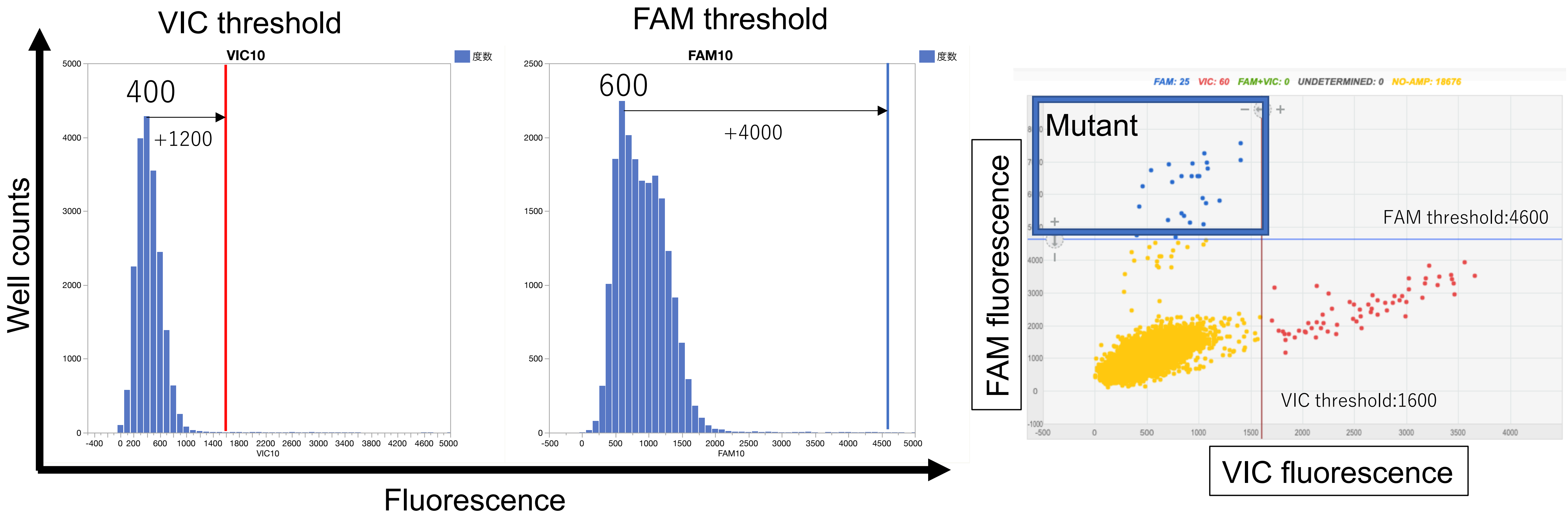

Mode: 400
VIC threshold:
Mode + 1200 = 1600

Mode: 600
FAM threshold:
Mode + 4000 = 4600

Supplementary **Fig 2**

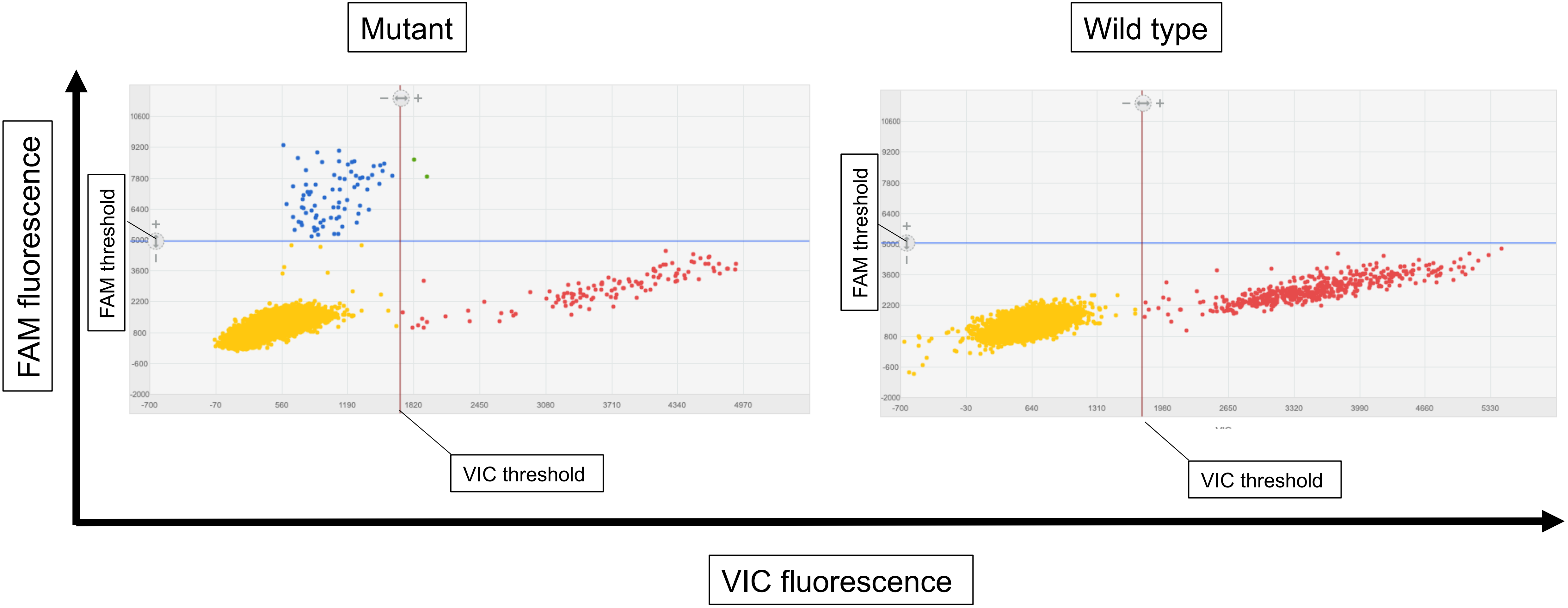

Supplementary **Fig 3a**: MRI imaging

FLAIR

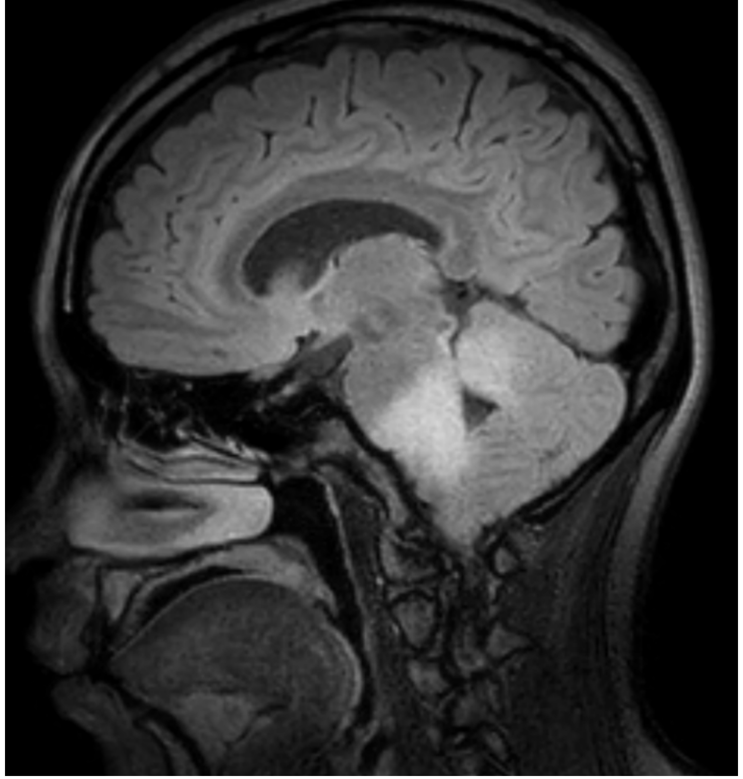

GdT1WI

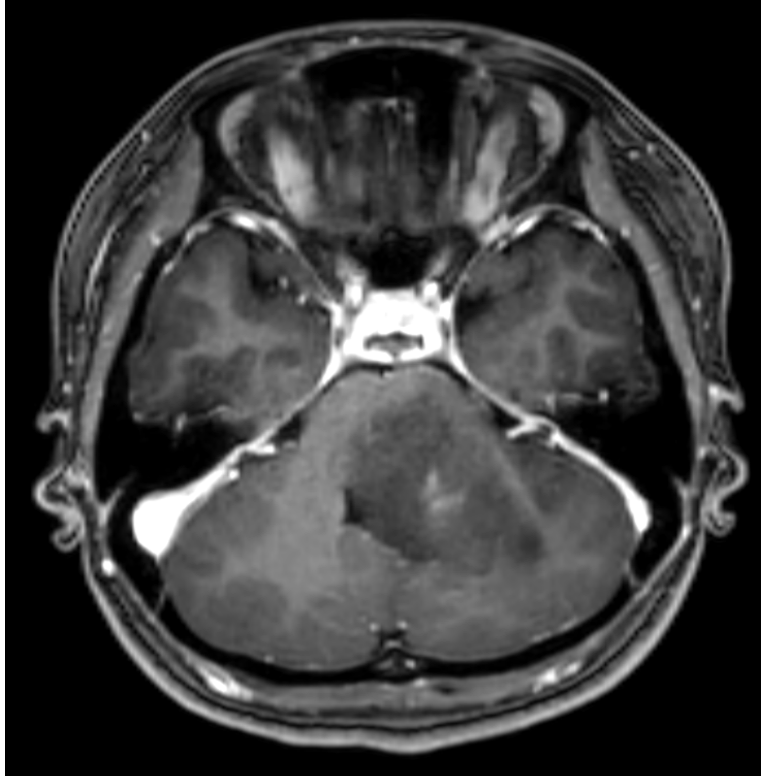

Pathological finding

Supplementary **Fig 3b**

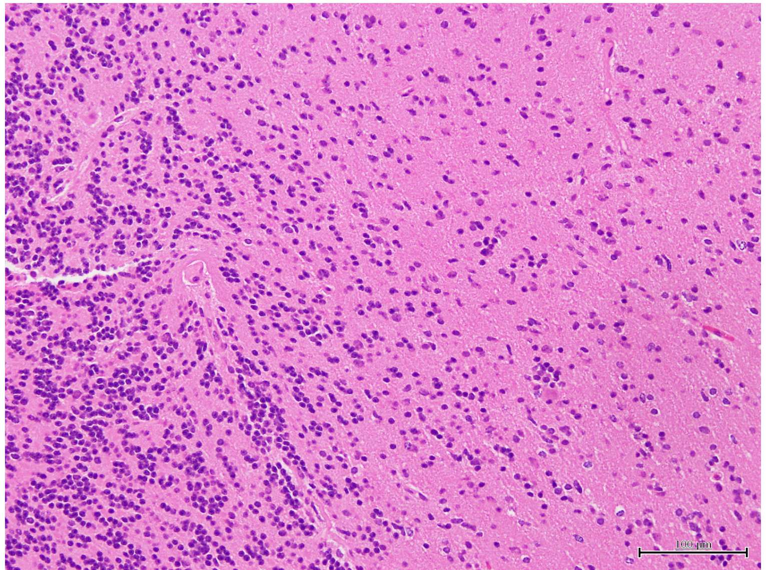

Supplementary **Fig 3c**

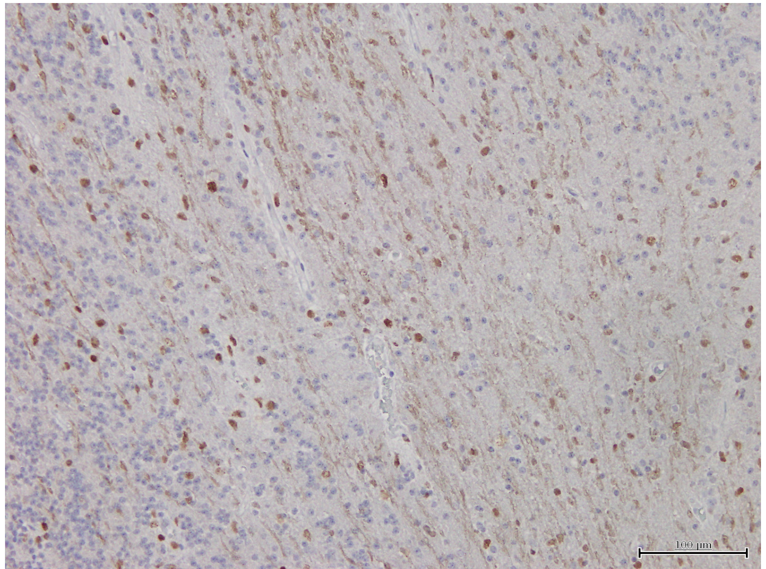

Supplementary **Fig 3d**:
Tumor DNA

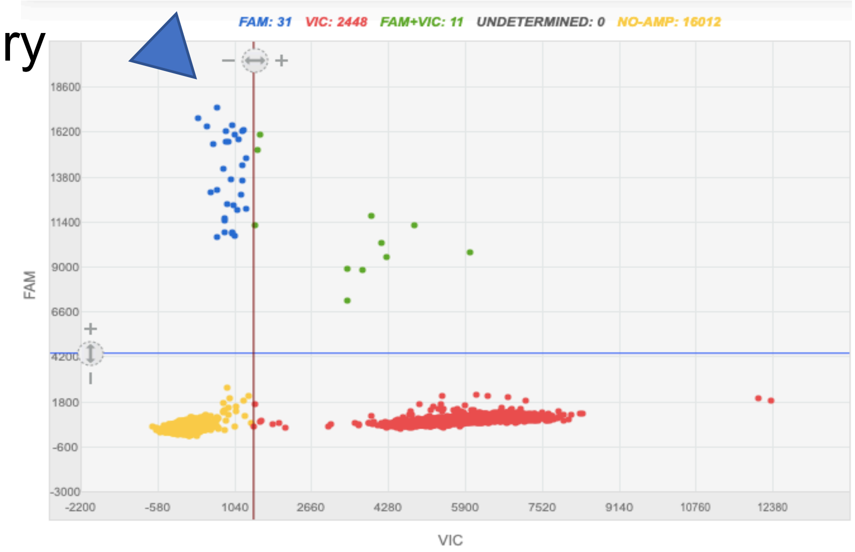

Digital PCR: positive

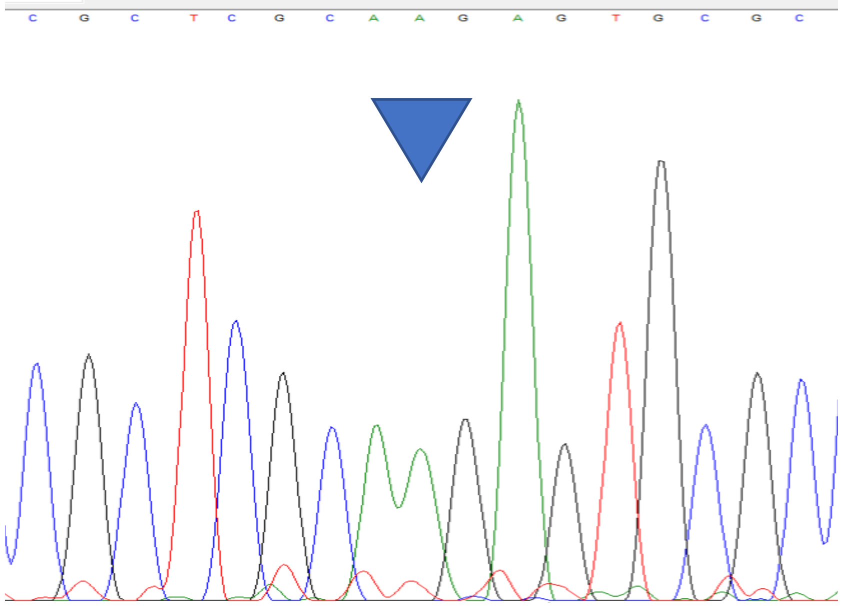

Sanger sequence: wild type

Supplementary **Fig 3e**:
cfDNA in CSF

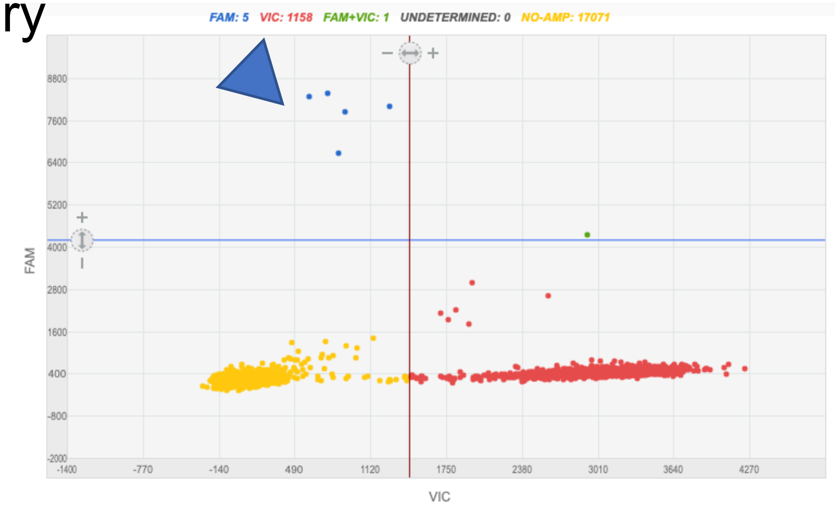

Intracranial CSF

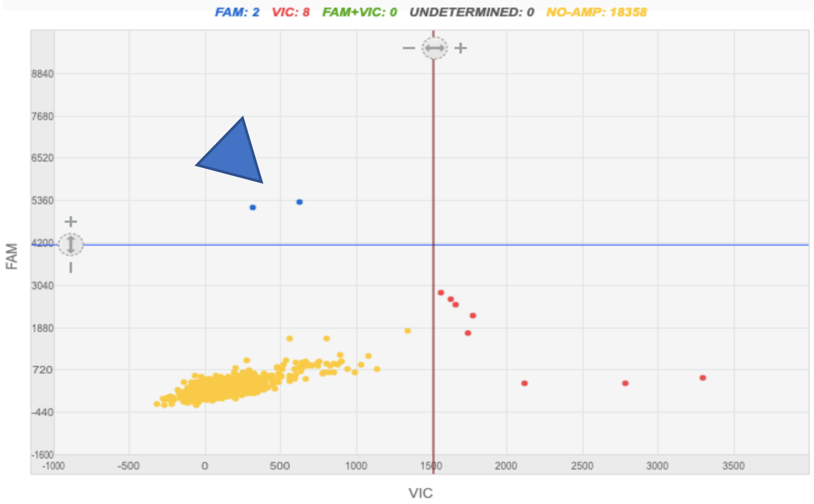

Lumbar puncture CSF
